# Using Insurance Claims Data to Estimate Blastomycosis Incidence, Vermont, USA, 2011–2020

**DOI:** 10.3201/eid3002.230825

**Published:** 2024-02

**Authors:** Brian F. Borah, Paul Meddaugh, Veronica Fialkowski, Natalie Kwit

**Affiliations:** Centers for Disease Control and Prevention, Atlanta, Georgia, USA (B. Borah);; Vermont Department of Health, Burlington, Vermont, USA (B. Borah, P. Meddaugh, N. Kwit);; Green Mountain Care Board, Montpelier, Vermont, USA (V. Fialkowski)

**Keywords:** blastomycosis, Blastomyces, fungi, respiratory infections, insurance claims, incidence, Vermont, United States

## Abstract

The epidemiology of blastomycosis in Vermont, USA, is poorly understood. Using insurance claims data, we estimated the mean annual blastomycosis incidence was 1.8 patients/100,000 persons during 2011–2020. Incidence and disease severity were highest in north-central counties. Our findings highlight a need for improved clinical awareness and expanded surveillance.

Blastomycosis is a rare but potentially fatal fungal disease caused by *Blastomyces* spp., a group of thermally dimorphic environmental mycoses found in moist soil and decaying organic matter. Human illness most often results in pulmonary conditions but can involve any organ system; clinical manifestations range from subclinical infection to life-threatening disease (*1*). Associated illness and death rates are high; among symptomatic persons, hospitalization rates are 57%–69% (*2*–*5*) and death rates 4%–22% (*1*).

Epidemiology of blastomycosis in the United States is poorly understood. Geographic areas of the United States that have historically been considered endemic, based largely on sporadic case reports and a few documented outbreaks, include midwestern, south-central, and southeastern regions of the country, particularly adjacent to the Ohio and Mississippi Rivers, Great Lakes, and St. Lawrence Seaway. In those areas, statewide annual incidence rates are ≈0.2–2.0 cases/100,000 persons (*1*–*3*). However, blastomycosis is not a nationally notifiable disease, and public health surveillance is limited to just 5 states: Arkansas, Louisiana, Michigan, Minnesota, and Wisconsin. The true burden of blastomycosis elsewhere is unknown. 

Recent studies suggest incidence in the United States, particularly in the northeastern region, might be greater than previously understood (*6*–*9*). To assess the epidemiology of blastomycosis in the northeastern state of Vermont, we used insurance claims data and vital records to describe case-patient demographics, hospitalization rates, deaths, annual incidence, and geographic distribution of disease. This activity was reviewed by the Centers for Disease Control and Prevention and conducted consistent with applicable federal law and agency policy. Activity was determined to meet the requirements of public health surveillance as defined in 45 CFR 46.102(l)(2). 

## The Study 

The Vermont Health Care Uniform Reporting and Evaluation System (VHCURES) is an all-payer health insurance claims database managed by the Green Mountain Care Board (Montpelier, Vermont, USA). VHCURES includes insurance claims data from medical, dental, and pharmacy encounters for all Medicare and Medicaid recipients and ≈75% of Vermont residents with commercial insurance. For this retrospective cohort analysis, we used VHCURES to identify all patients who received a primary or secondary diagnosis code of 116.0 (International Classification of Disease [ICD], 9th Edition, Clinical Modification) or B40.X (ICD, 10th Edition, Clinical Modification) for blastomycosis during a 2011–2020 medical encounter. VHCURES excludes personally identifiable information but does provide patient-level information including age, sex, insurance type (Medicare, Medicaid, or commercial), county of residence, and hospitalization status. Race and ethnicity data were unavailable. We identified blastomycosis-attributable deaths from Vermont vital records and calculated incidence rates (cases/100,000 persons) using state- and county-level census estimates.

**Figure 1:**
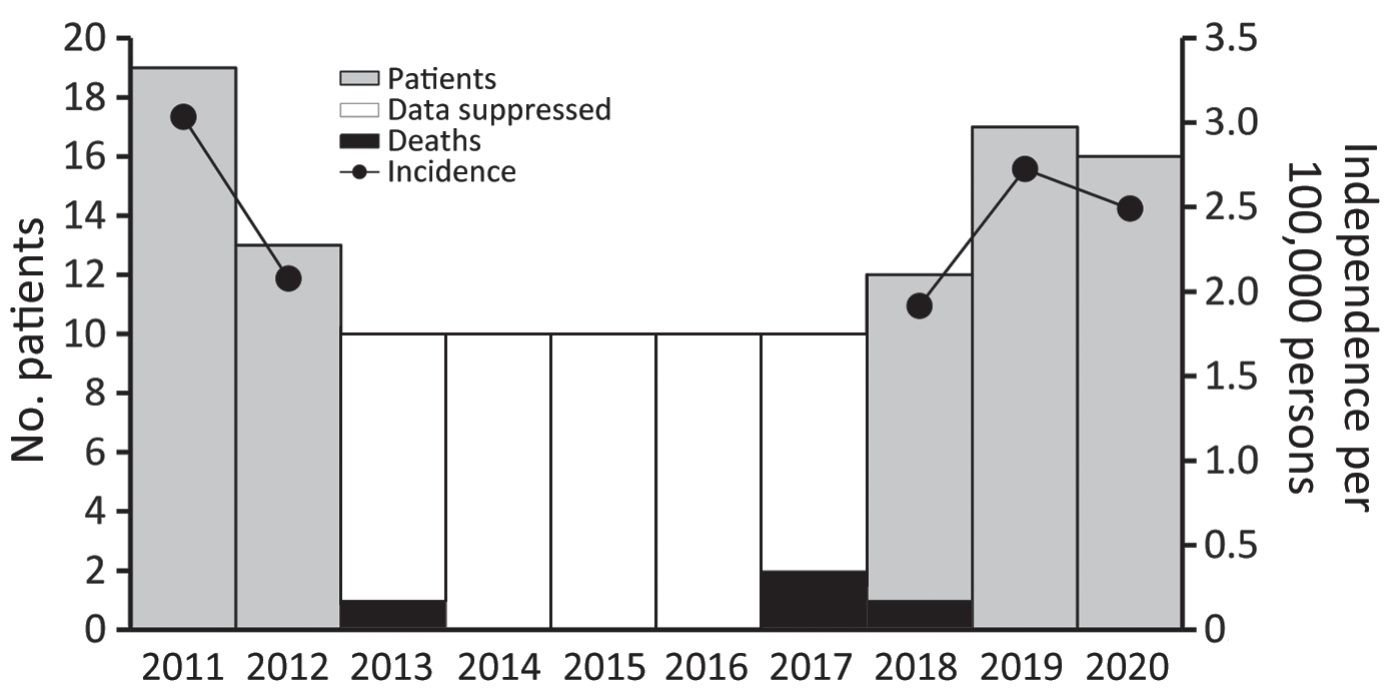
Numbers of patients with blastomycosis, attributable deaths per year, and annual incidence (cases/100,000 population) in Vermont, USA, 2011–2020. Results are suppressed for years with ≤10 patients, in accordance with the Green Mountain Care Board data use agreement for the Vermont Health Care Uniform Reporting and Evaluation System.

We identified 114 patients with blastomycosis diagnosed during 2011–2020, a median of 10.5 (range 6–19 cases) cases/year. Most case-patients were male (67; 59%), and median age at first diagnosis was 55 years (range: 0–89 years) (Table). At the time of first diagnosis, 48 (42%) patients had commercial insurance, 42 (37%) Medicare, and 24 (21%) Medicaid. Mean annual statewide incidence of blastomycosis was 1.8 cases/100,000 persons; incidence among male residents (2.2/100,000 population) was greater than that among female residents (1.5/100,000 population). The highest annual incidence, 3.0/100,000 persons, occurred in 2011, followed by 2019 (2.7/100,000 population) and 2020 (2.5/100,000 population) (Figure 1). 

**Table:** Characteristics of patients with blastomycosis, Vermont, USA, 2011–2020*

Characteristic	All patients	Hospitalized patients	Deaths
Total	114 (100)	34 (30)	4†
Sex			
M	67 (59)	20 (59)	1 (25)
F	47 (41)	14 (41)	3 (75)
Insurance type			
Commercial	48 (42)	≤10†	NA
Medicare	42 (37)	19 (56)	NA
Medicaid	24 (21)	≤10†	NA
Age category, y			
0–19	≤10‡	≤10†	0
20–39	21 (18)	≤10†	0
40–59	40 (35)	11 (32)	2 (50)
60–79	43 (38)	12 (35)	1 (25)
≥80	≤10‡	≤10†	1 (25)
Median age, y (range)	55 (0–89)	56 (3–89)	59 (45–90)

*Values are no. (%) except as indicated. †Deaths were identified from different data source than patients and hospitalizations; death rate cannot be calculated. ‡In accordance with the Green Mountain Care Board data use agreement for the Vermont Health Care Uniform Reporting and Evaluation System, all results with a patient count ≤10 must be suppressed.

Thirty-four (30%) patients had >1 blastomycosis-associated hospitalization during the study period. Median age of hospitalized patients at time of diagnosis was 56 (range 3–89) years; 20 (59%) were male and 19 (56%) covered by Medicare. Risk for hospitalization was similar between male and female patients (risk ratio 1.00, 95% CI 0.57–1.78). According to vital records data, 4 deaths were attributed to blastomycosis, a mean annual death rate of 0.06/100,000 population. Three deaths were among female patients, at a median age of 59 years.

**Figure 2:**
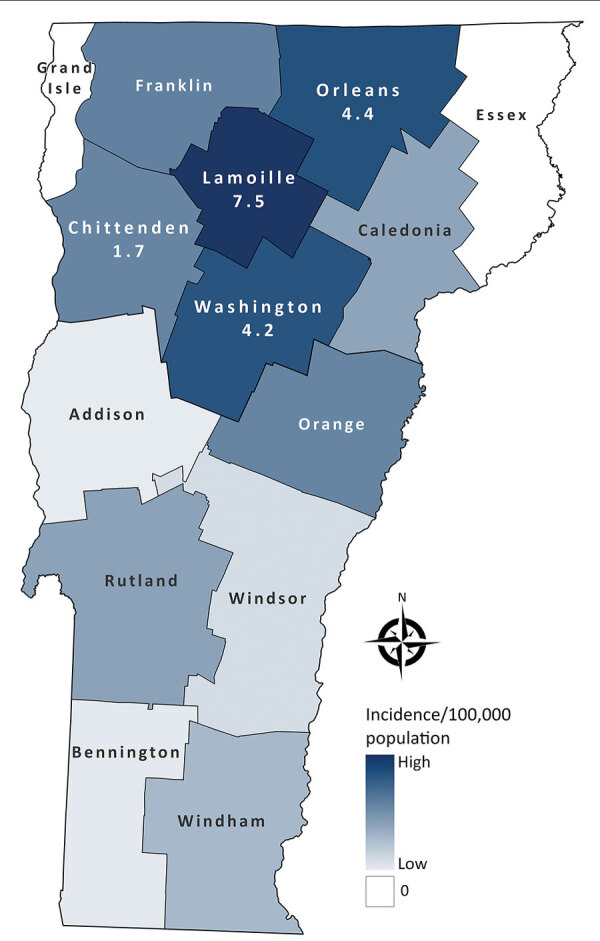
Geographic distribution of blastomycosis cases by county, Vermont, USA, 2011–2020. Numbers indicate incidence rates (cases/100,000 population) for counties with the highest incidence.

Of Vermont’s 14 counties, 3 counties in north-central Vermont (Lamoille, Orleans, and Washington) had the highest mean annual incidences (Figure 2). Although the populations of those counties represent only 18% of the state population, 49% of all case-patients and 65% of hospitalized patients resided in those counties. Case-patients in those counties were ≈2 times as likely to be hospitalized with blastomycosis as patients residing elsewhere (risk ratio 1.90, 95% CI 1.04–3.46).

Although Vermont has historically not been considered an area with high relative incidence of blastomycosis, an estimated mean annual incidence of 1.8 case-patients/100,00 population suggests otherwise. That incidence is greater than the mean annual incidences during 1987–2017 in 4 of 5 states that mandate reporting of blastomycosis (Arkansas, Louisiana, Michigan, and Minnesota, but not Wisconsin) (*2*); incidence in Vermont was greater than in all 5 of those states in 2019 (*3*). Missouri (*10*), Mississippi (*11*), and Illinois (*12*), also located within known endemic areas, reported mean annual incidences of 0.2–1.3/100,000 population for differing intervals during 1979–2018. Although differences in surveillance methods and case definitions among states make direct comparisons difficult, Vermont’s burden of blastomycosis appears comparable to, and perhaps higher than, most states that have published blastomycosis incidences. 

Consistent with other published studies, our results demonstrate that blastomycosis was more common in adults and male patients (*2*,*3*). We also found that disease incidence and hospitalization rates were greatest in north-central Vermont, a finding supported by clinician reports and at least 1 publication (*6*). Explanations for regional clustering and high statewide incidence in Vermont are unclear. Outdoor activities, climate, geographic features, and soil characteristics have all been associated with heterogeneity of blastomycosis distribution elsewhere (*1*,*2*,*4*,*13*). Like hyperendemic regions of Wisconsin, Vermont is rich in acidic spodosol soil (*14*), which is thought to support *Blastomyces* spp. growth (*2*). Although the 3 counties with the highest incidence in Vermont do not share a common waterway, proximity to waterways generally has been associated with disease (*1*,*13*). Future studies, including animal, environmental, and ecologic niche models (*13*), could further characterize these and other risk factors in Vermont.

Among limitations in our study, blastomycosis diagnoses are commonly delayed or missed in clinical practice because of low clinical suspicion and nonspecific diagnostic tests (*1*), and laboratory-confirmed diagnoses can be missed by ICD-based queries (*10*). Next, claims data are used primarily for administrative purposes and thus have inherent limitations for public health surveillance, including coding errors and disease misclassification. We might also have undercounted diagnoses among the minority of the Vermont population who did not have claims submitted to VHCURES. Moreover, given inherent complexities in VHCURES data which limit accurate estimations of total annual VHCURES enrollees, we used census estimates of state population for incidence denominators. Those limitations likely resulted in an underestimation of blastomycosis incidence. However, other limitations might have posed some risk of overestimation. We might have included patients who were diagnosed with blastomycosis before 2011 if they had a follow-up encounter associated with the diagnosis during 2011–2020. In addition, we were unable to validate ICD-based diagnoses with external laboratory or clinical data. Our methods possibly captured mild illnesses better than passive surveillance, which is biased toward severe cases (*15*), which might explain why the hospitalization rate (30%) in our study was lower than rates elsewhere (*2*–*5*). Finally, we used county of residence of case-patients to describe geographic distribution of the disease; we could not determine whether fungal exposures were travel-associated. 

## Conclusions 

Our findings, based on the most comprehensive assessment of blastomycosis in Vermont to date, align with a growing body of evidence suggesting that the burden of endemic blastomycosis is greater than commonly appreciated (*6*–*9*). These results challenge routine assumptions about the epidemiology and ecology of this disease and reflect a need for future studies. Clinicians should consider blastomycosis in patients with compatible signs and symptoms. Standardized surveillance could also improve our understanding of exposures, risk factors, and clinical outcomes. 
